# An increase in *SNHG5* expression is associated with poor cancer prognosis, according to a meta-analysis

**DOI:** 10.1186/s40001-024-01745-3

**Published:** 2024-03-12

**Authors:** Qiang Huang, Yi-gui Xia, Yong-jian Huang, Hai-feng Qin, Qun-xian Zhang, Chun-feng Wei, Wu-ru Tang, Yuan Liao

**Affiliations:** 1 Department of Laboratory Medicine, Hechi Hospital Affiliated to Youjiang Medical University for Nationalities, the People’s Hospital of Hechi, Hechi, 547000 Guangxi China; 2 Department of Nuclear Medicine, Hechi Hospital Affiliated to Youjiang Medical University for Nationalities, the People’s Hospital of Hechi, Hechi, 547000 Guangxi China; 3 Department of Oncology, Hechi Hospital Affiliated to Youjiang Medical University for Nationalities, the People’s Hospital of Hechi, Hechi, 547000 Guangxi China

**Keywords:** lncRNA, *SNHG5*, Cancer, Prognosis, Meta-analysis

## Abstract

**Background:**

He long noncoding RNA small nucleolar host RNA 5 (*SNHG5*) is highly expressed in many cancers, and there is a notable correlation between the elevated expression of *SNHG5* and survival outcome in cancer patients. The objective of this study was to conduct a meta-analysis to evaluate the correlation between *SNHG5* expression and the clinical outcome of cancer patients.

**Methods:**

Six relevant electronic databases were exhaustively searched, and, depending on the inclusion and exclusion criteria, appropriate literature was obtained. The Newcastle-Ottawa Scale (NOS) score was utilized to evaluate the quality of the research for every article included, and pertinent data from each study were carefully extracted. Hazard ratios (HRs), odds ratios (ORs) and 95% confidence intervals (CIs) were combined to explore the association of *SNHG5* expression levels with cancer prognosis, and sensitivity analyses and assessments of publication bias were also conducted to investigate any possibility in the publication of the studies.

**Results:**

Eleven studies encompassing 721 patients were ultimately collected. When combined, the hazard ratios (HRs) revealed a substantial direct correlation between elevated *SNHG5* expression and an unfavourable prognosis for cancer patients (HR = 1.90, 95% CI 0.87–4.15); however, the correlation did not reach statistical significance. Furthermore, high *SNHG5* expression was predictive of advanced TNM stage (OR: 1.988, 95% CI 1.205–3.278) and larger tumour size (OR: 1.571, 95% CI 1.090–2.264); moreover, there were nonsignificant relationships between *SNHG5* expression and DM (OR: 0.449, 95% CI 0.077–2.630), lymph node metastasis (OR: 1.443, 95% CI 0.709–2.939), histological grade (OR: 2.098, 95% CI 0.910–4.838), depth of invasion (OR: 1.106, 95% CI 0.376–3.248), age (OR: 0.946, 95% CI 0.718–1.247) and sex (OR: 0.762, 95% CI 0.521–1.115).

**Conclusion:**

*SNHG5* expression is typically increased in the majority of tumour tissues. Elevated *SNHG5* expression may indicate poor prognosis in cancer patients. Therefore, *SNHG5* is a promising potential therapeutic target for tumours and a reliable prognostic biomarker.

## Introduction

Cancer has caused social and public problems that cannot be ignored, with huge economic losses and mental burdens to people all over the world every year [1, 2]. Based on the 2021 Cancer Statistics Report, the numbers of individuals newly diagnosed with cancer and dying from cancer in 2020 will be approximately 19.84 million and 10 million, respectively [3, 4]. Despite the well-being and satisfaction of cancer patients having improved to a certain extent alongside advancements in molecular biology technology and medical care, the 5-year survival rate for cancer patients has remained unsatisfactory [5, 6]. The main reason is that classic treatment methods such as chemotherapy, radiation therapy, hormone therapy and targeted therapeutics are already in their prime, and it is difficult for these methods to improve survival [7–10]. Therefore, novel therapeutic targets aimed at improving the prognosis of cancer patients are urgently needed [11–13].

Over the past few years, the employment of high-throughput sequencing techniques and advancements in molecular biology have gradually revealed an increasing number of genes that are intricately linked to cancer. Additionally, a growing body of evidence has confirmed the involvement of numerous noncoding RNAs in the initiation and progression of cancer [14, 15]. Noncoding RNAs are a class of small molecular compounds that lack the ability to encode proteins [16–18]. They are called the "useless product" of genetic material and account for 95–98% of human genetic material [19]. Although noncoding RNAs do not perform the biological function of encoding proteins, there is substantially more evidence that noncoding RNAs can exert their control over the biological behaviour of cells by influencing the expression of cell-coding genes at various levels, encompassing gene transcription, post-transcriptional translation, and epigenetic regulation [20–22]. Noncoding RNAs can affect the proliferation, invasion and apoptosis of tumour cells, thereby affecting the progression of tumours. For example, Professor Yang showed that low expression of lncRNA-BANCR can significantly stimulate the growth and motility of lung cancer cells and suppress programmed cell death, thereby contributing to the initiation and progression of lung cancer [23]. Professor Yan stated that high expression of lnc-SNHG6 can significantly suppress the programmed cell death (apoptosis) of gastric cancer cells, stimulate their proliferation, migration, and invasion, and exhibit a strong correlation with an unfavourable prognosis [24]. Noncoding RNA are being considered by more and more researchers as promising targets and prognostic indicators for future cancer therapies.

Lnc-SNHG5 is a category of low-molecular-weight compounds characterized by more than 200 nucleotide units that lack protein coding ability [25]. An increasing number of studies have shown that *SNHG5* is differentially expressed across various tumour cell types and is implicated in the aetiology and progression of diverse cancers [26, 27]. For example, Professor Wei showed that *SNHG5* is abundantly expressed in oesophageal cancer tissues and that elevated *SNHG5* levels can enhance the proliferative and migratory potential of oesophageal squamous cell carcinoma (ESCC) cells, suppress cellular apoptosis, and consequently promote the progression of ESCC [28]. Professor Kang reported that *SNHG5* is overexpressed in lung cancer, where its high expression facilitates the movement and infiltration of lung cancer cells while suppressing their apoptosis. Furthermore, numerous studies have increasingly indicated a significant association between elevated *SNHG5* expression and poor lung cancer prognosis [29]. Inconsistencies across different studies involving relatively small numbers of patients have made conclusions questionable; even though some outcome indicators have suggested that *SNHG5* is a poor prognostic marker, the differences did not reach statistical significance. Hence, the objective of this investigation was to conduct a meta-analysis to investigate the potential correlation between the expression level of *SNHG5* and cancer prognosis.

## Materials and methods

### Inclusion of appropriate literature

Utilizing the reporting guidelines established by the Preferred Reporting Items for Systematic Reviews and Meta-Analyses (PRISMA) as the foundation for our reporting format, a comprehensive search was performed by browsing relative databases such as PubMed, Embase, the Web of Science, the Cochrane Library, Google Scholar, the China National Knowledge Infrastructure (CNKI) and the Wanfang Database from the establishment of the database to January 1, 2023. The retrieval strategies used in this study were as follows: “Small nucleolar RNA host gene 5” OR “lncRNA Small nucleolar RNA host gene 5” OR “lncRNA SNHG5” OR “SNHG5” OR “lncSNHG5”) AND “cancer” OR “carcinoma” OR “prognosis” OR “survival” OR “survival prognosis.” We also consulted the references of the included publications in detail to obtain useful and appropriate publications.

### Inclusion and exclusion criteria

Publications that fulfilled the following criteria were deemed appropriate for inclusion in this meta-analysis: (1) the fundamental purpose of the literature review was to evaluate the relationship between *SNHG5* expression levels and cancer prognosis; (2) patients were divided into two distinct groups based on their expression level (high or low); (3) the research subjects were limited to human patients; (4) provided enough raw data to be extracted. Studies with the following characteristics were considered unsuitable for inclusion in this meta-analysis: (1) lacked sufficient data; (2) the participants were animals; (3) literature reviews, meta-analyses, case reports, conference summaries, and research not officially published; (4) non-English language publications.

### NOS score of included studies

The Newcastle-Ottawa Scale (NOS) score, which includes eight items, was utilized for evaluating the overall quality of the studies included [30]. Two researchers meticulously and independently assessed the quality of every included article, taking into consideration the aforementioned NOS score. If the evaluation results were not consistent, an agreement was reached through discussion or a third researcher was consulted for discussion and confirmation. The total NOS score ranged from 0 to 9. Articles with a score of 6 or more were considered high-quality documents and suitable for inclusion in this study. Articles with a score less than 6 points were considered low-quality studies and were excluded from this investigation.

### Data extraction

The useful raw data were obtained by two researchers independently, and the detailed information included the name of the primary author, the year the article was published, the total number of patients involved, the reference gene, the cutoff value, and the country to which the patient belonged. We also extracted the main outcome indicators, including overall survival (OS) and relapse-free survival (RFS). Furthermore, secondary outcome indicators, including TNM stage, lymph node metastasis (LNM) status, distant metastasis(DM) status, tumour dimensions, histological grade, depth of infiltration, chronological age and sex, were obtained. If the survival data provided in the publication included multivariate analysis and univariate analysis, only multivariate analysis was obtained; for example, if the study only included the survival curve, then the detailed survival data were obtained utilizing Engauge Digitizer V 4.1 software, and the study included the time-dependent survival rates of both the high- and low-expression groups of *SNHG5* [31].

### Statistics and analysis

RevMan V 5.4 software and STATA V 12.0 software were used to perform the statistical analysis of this meta-analysis. Patients were categorized into either a high-expression group or a low-expression group according to the original literature reports. The combination of the odds ratio (OR) with 95% confidence interval (CI) was used to evaluate the associations between *SNHG5* expression and TNM stage, LNM, DM, tumour size, etc. The combination of the hazard ratio (HR) and 95% confidence interval (CI) was used to evaluate the relationship between *SNHG5* expression and cancer prognosis, including OS and RFS. If *I*^*2*^ (I-square) < 50% and *P* > 0.05, the result was considered to indicate insignificant heterogeneity, and a fixed effects model was used. If *I*^*2*^ > 50% and *P* < 0.05 were significant, significant heterogeneity was considered. A random effects model was used, and subgroup analysis was conducted based on cancer type (digestive system and nondigestive tract), number of patients (fewer than 60 patients and no less than 60 patients), follow-up month (fewer than 60 and no less than 60), cutoff value (mean and median), and data analysis method (multivariate analysis and univariate analysis). Sensitivity analysis was conducted using STATA software to assess whether the results of individual studies had a significant impact on the overall findings. Additionally, Begg's analysis was employed to detect any significant publication bias in the original study.

## Results

### Characteristics of the enrolled publications

**Fig. 1:**
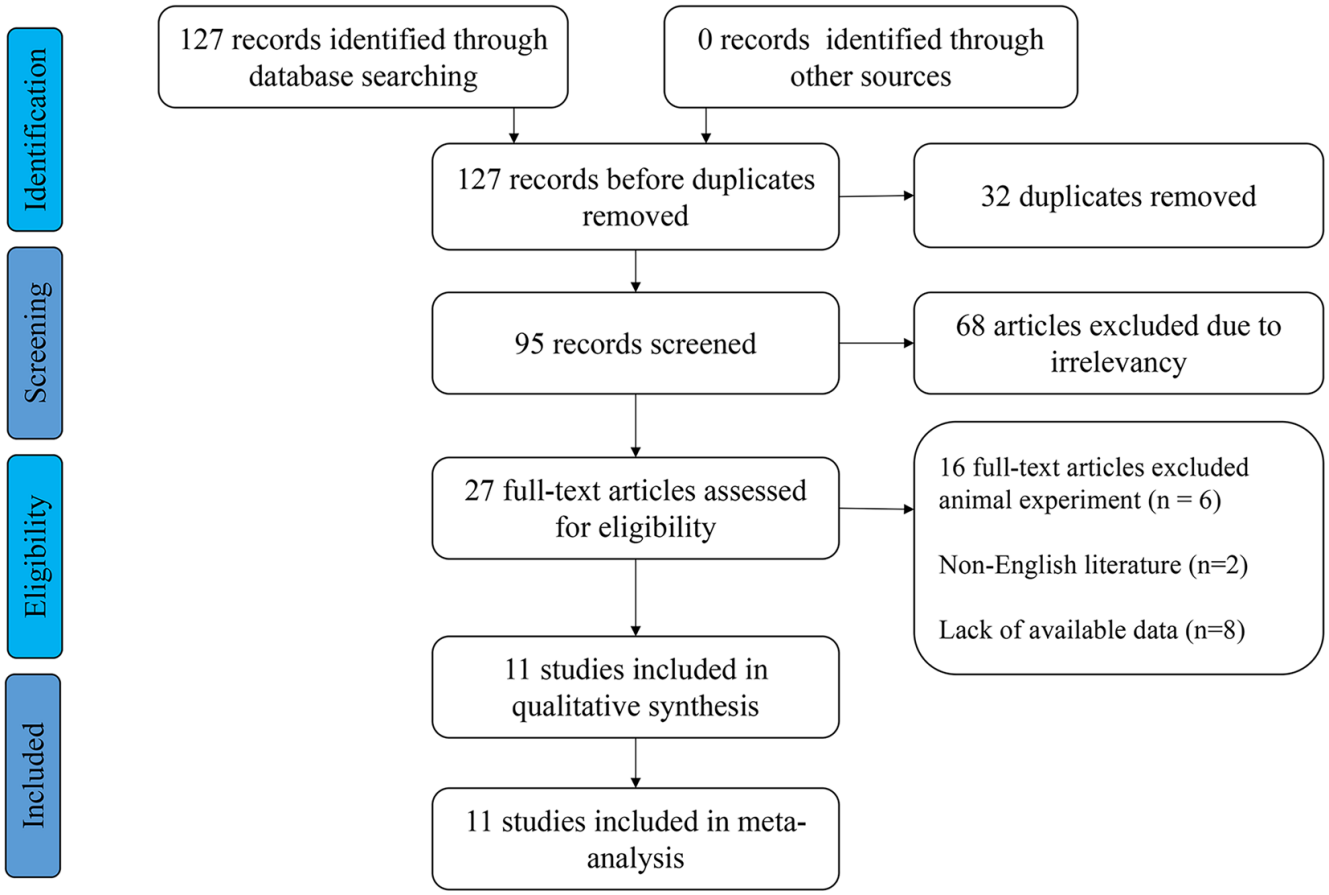
Literature search and inclusion process

After the systematic and detailed searches, 127 publications were initially obtained, 32 duplicate documents were found, 68 articles were excluded for not exploring the relationship between *SNHG5* expression and cancer prognosis, 6 publications were excluded for involving animal experimentation, 8 articles lacked sufficient data, and 2 non-English papers were also excluded. Finally, 11 suitable investigations involving 721 patients were included in this meta-analysis (Fig. 1). The cancer types included bladder cancer [32], hepatocellular carcinoma [33], cervical cancer [34], osteosarcoma [35], nasopharyngeal carcinoma [36], non-small cell lung cancer [29], oesophageal cancer [28], diffuse large B-cell lymphoma [37], and gastric cancer [38, 39]. All the patients were Chinese, and the number of patients in each study varied between 32 and 90; seven [7] papers provided survival data (Table 1). According to the NOS score, the enrolled studies ranged from 7 to 8, indicating that all the papers were suitable for enrolment and analysis (Table 2).

**Table 1:** Basic features of the publications included in this meta-analysis (*n* = 11)

First author	Year	Cancer type	Number of patients	SNHG5 expression	Referrence gene	Cut-off value	Survival analysis	HR statistics	Hazard ratios (95%CI)	Analysis method	Follow-up (month)	NOS score
Ma ZP	2018	Bladder cancer	67	Upregulated	GAPDH	Mean	OS	Survival curve	2.82 (1.13–7.01)	Univariate analysis	60	8
Li YR	2018	HCC	48	Upregulated	GAPDH	Median	OS	Paper	4.74 (1.350–6.640)	Multivariate analysis	36	8
							RFS		3.690 (1.229–11.082)	Multivariate analysis	36	8
Zhang LY	2021	CC	40	Upregulated	GAPDH	Median	Not reported	–	–	–	–	7
Wang ZW	2018	Osteosarcoma	32	Upregulated	GAPDH	Median	OS	Survival curve	7.52 (1.66–34.1)	Univariate analysis	36	7
Liu DT	2020	Nasopharyngeal carcinoma	64	Upregulated	GAPDH	Median	OS	Survival curve	1.57 (0.92–2.68)	Univariate analysis	60	7
Wei SS	2021	Esophageal cancer	77	Upregulated	GAPDH	Median	OS	Survival curve	0.42 (0.22–0.8)	Univariate analysis	60	8
Xing XJ	2022	DLBCL	90	Upregulated	GAPDH	Mean	Not reported	–	–	–	–	7
Zhao L	2016	GC	87	Upregulated	GAPDH	Median	OS	Survival curve	0.59 (0.32–1.09)	Univariate analysis	48	7
Li XY	2021	GC	50	Downregulated	β-actin	Median	OS	Paper	4.890 (2.125–14.633)	Multivariate analysis	15	8
Ying XY	2019	AML	80	Upregulated	GAPDH	Mean	OS	Survival Curve	2.06 (1.19–3.59)	Univariate analysis	60	7
Kang SY	2023	NSCLC	86	Upregulated	GAPDH	Median	Not reported	–	–	–	–	7

No.: number; NSCLC: non-small cell lung cancer; HCC: hepatocellular carcinoma; DLBCL: diffuse large B cell lymphoma; CC: Cervical cancer; GC: gastric cancer; NA: not available; qRT-PCR: quantitative reverse transcription-polymerase chain reaction; AML: acute myeloid leukemia; HR: hazard ratio; NOS: Newcastle–Ottawa scale; CI confidence interval; GAPDH: glyceraldehyde-3-phosphate dehydrogenase

**Table 2:** Quality assessment of eligible studies Newcastle–Ottawa scale (NOS) score

Author	Selection	Representativeness of the cases	Selection of Controls	Definition of Controls	Comparability	Outcome	Same method of ascertainment	Non-Response rate	Total
	Adequate of case definition				Comparability of cases and controls	Ascertainment of exposure			
Ma ZP 2018	*	*	*	*	*	*	*	*	8
Li YR 2018	*	*	*	*	*	*	*	*	8
Zhang LY 2021	*	*	*	*	*	*	*	–	7
Wang ZW 2018	*	*	*	*	*	*	*	–	7
Liu DT 2020	*	*	*	*	*	*	*	–	7
Wei SS 2021	*	*	*	*	*	*	*	*	8
Xing XJ 2022	*	*	*	*	–	*	*	*	7
Zhao L 2016	*	*	*	*	*	*	*	–	7
Li XY 2021	*	*	*	*	*	*	*	*	8
Ying XY 2019	*	*	*	*	*	*	*	–	7
Kang SY 2023	*	*	*	*	*	*	*	–	7

“*” indicates that the original article meets this entry based on the NOS score, and “_” indicates that the original article does not meet this entry. For example, the article of Zhang LY 2021 miss the The follow-up time, so the module of "non-response rate" of NOS score is also missing, and therefore deducted pointss

### The correlation between *SNHG5* expression and survival outcome

**Fig. 2:**
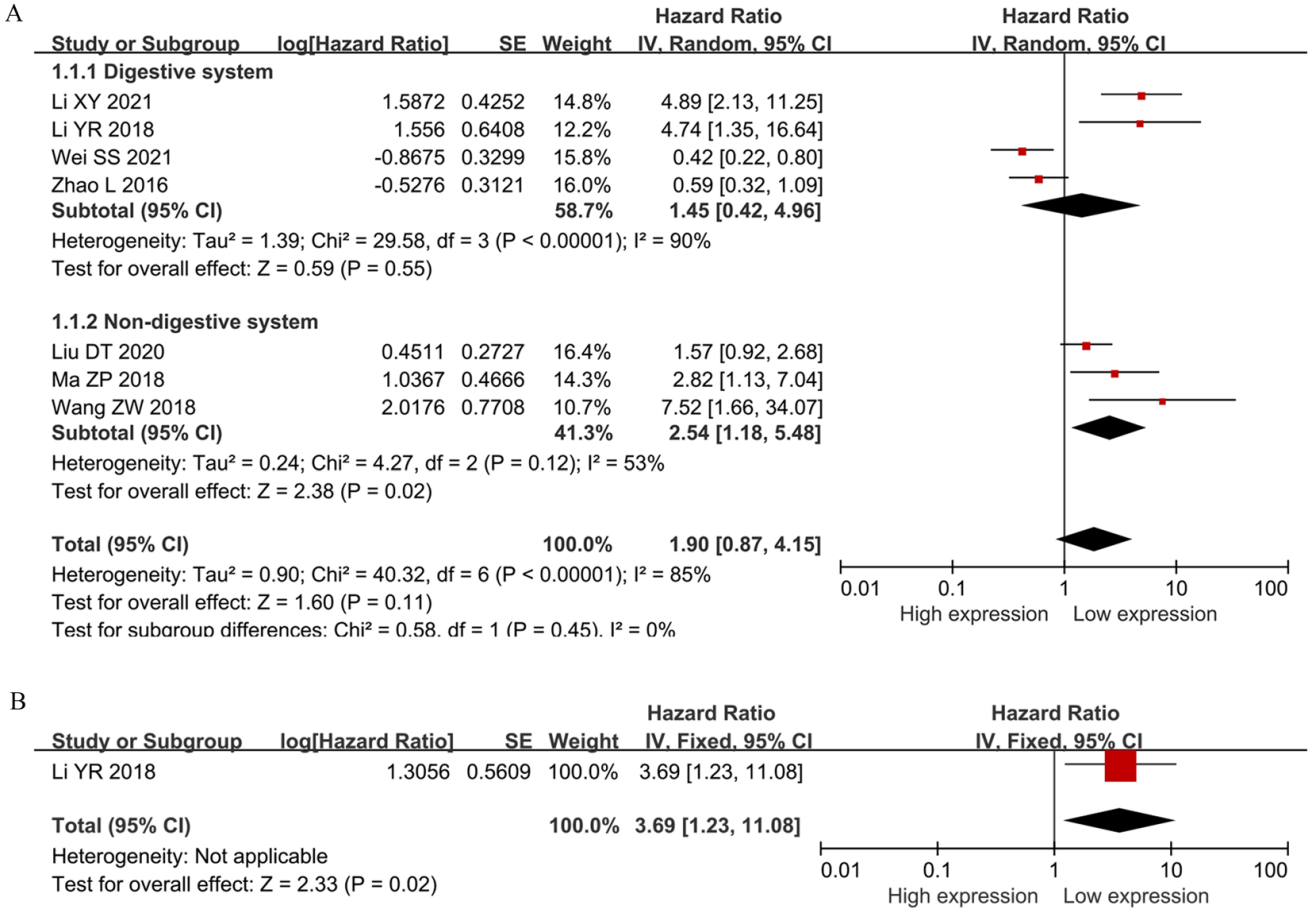
Forest plot of SNHG5 expression and survival outcome in cancers. **A** OS; **B** DFS

Seven studies involving a total of 425 patients were obtained to evaluate the correlation between *SNHG5* expression and cancer prognosis. A positive correlation was revealed between elevated *SNHG5* expression and poor survival outcome (HR = 1.90, 95% CI 0.87–4.15) (Fig. 2), but the correlation was not statistically significant. Considering the inconsistent results of *SNHG5* expression in patients with different tumour types, a subgroup analysis was conducted, taking into account cancer type, sample size, cutoff value, follow-up duration, hazard ratio (HR) statistics, and analysis method. The findings indicated that elevated *SNHG5* expression was associated with a poorer cancer prognosis, specifically in patients with nondigestive system cancers (HR = 2.54, 95% CI 1.18–5.48); multivariate analysis was also performed (HR = 4.84, 95% CI 2.42–9.70), and the mean cutoff value was 2.82, 95% CI 1.13–7.04. Moreover, there was an insignificant relationship between *SNHG5* expression and OS in the digestive system subgroup according to cancer type (HR = 1.45, 95% CI 0.42–4.96); univariate analysis was also performed (HR = 1.31, 95% CI 0.58–2.99), and the median cutoff value was 1.79, 95% CI 0.74–4.30 (Table 3). In addition, the results of one original study demonstrated that increasing *SNHG5* expression might contribute to poor DFS (hazard ratio (HR): 3.690, 95% CI 1.229–11.082) (Table 3).

**Table 3:** Pooled HRs of overall survival of patients with increased SNHG5 expression

Subgroup analysis	Number of studies	Number of patients	Pooled HR(95%CI)		*I*^*2*^(%)	*P*-value	Model
			Fix effect model	Random effect model			
OS	7	425	1.33 (1.00–1.77)	1.90 (0.87–4.15)	85	< 0.00001	Random
Tumor type							
Digestive system	4	262	0.97 (0.67–1.41)	1.45 (0.42–4.96)	90	< 0.00001	Random
Non-digestive system	3	163	2.06 (1.32–3,20)	2.54 (1.18–5.48)	53	0.12	Random
Analysis method							
Univariate analysis	5	327	1.02 (0.75–1.40)	1.31 (0.58–2.99)	84	< 0.0001	Random
Multivariate analysis	2	98	4.84 (2.42–9.70)	4.84 (2.42–9.70)	0	0.97	Fixed
Cut-off value							
Mean	1	67	2.82 (1.13–7.04)	2.82 (1.13–7.04)	–	–	–
Median	6	358	1.23 (0.91–1.65)	1.79 (0.74–4.30)	87	< 0.00001	Random
HR statistics							
Paper	2	98	4.84 (2.42–9.70)	4.84 (2.42–9.70)	0	0.97	Fixed
Survival curve	5	327	1.02 (0.75–1.40)	1.31 (0.58–2.99)	84	< 0.0001	Random
Number of patients							
Not less than 60	4	295	0.93 (0.68–1.29)	0.99 (0.45–2.18)	83	0.0006	Random
Less than 60	3	130	5.23 (2.78–9.83)	5.23 (2.78–9.83)	0	0.87	Fixed
Follow-up (month)							
Not less than 60 month	4	295	1.11 (0.76–1.62)	1.19 (0.42–3.42)	86	0.0008	Random
Less than 60 month	3	130	1.70 (1.09–2.63)	2.95 (0.75–11.64)	87	< 0.0001	Random
DFS	1	48	3.690 (1.229–11.082)	3.690 (1.229–11.082)	–	–	–

The results were performed by the software of Revman version 5.4

OS: overall survival; Random: Random effects; Fixed: Fixed effects; directly: HR was extracted directly from the primary articles; indirectly: HR was extracted indirectly from the primary articles; CI confidence interval; *I*^2^: I-square

### The correlation between *SNHG5* expression and TNM stage

**Fig. 3:**
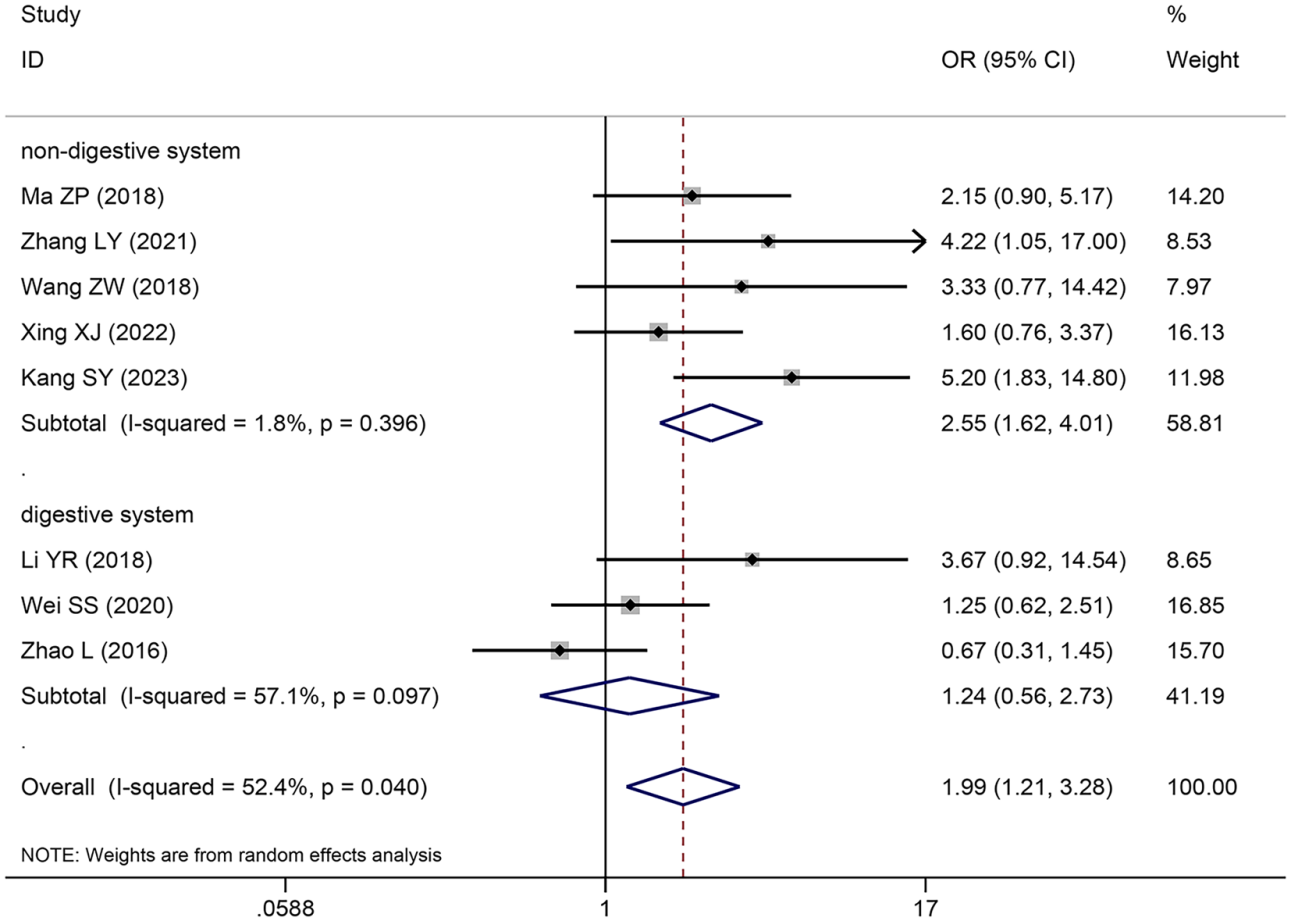
Forest plot of SNHG5 expression and TNM stage in cancers

Eight original studies comprising 527 patients were included in this study to explore the correlation between *SNHG5* expression and TNM stage. A pooled OR with 95% CI was used to determine the strong positive correlation between high *SNHG5* expression and advanced TNM stage (OR = 1.988, 95% CI 1.205–3.278) (Fig. 3). Due to inconsistencies in cancer types between different primary studies, subgroup analyses were also performed. The results of subgroup analysis demonstrated that increasing *SNHG5* expression predicted advanced TNM stage in the nondigestive system subgroup (OR = 2.617, 95% CI 1.686–4.061), and an insignificant correlation was observed in the digestive system subgroup (OR = 1.237, 95% CI 0.560–2.733) (Table 4).

**Table 4:** Pool effects of clinicopathologic characteristics in cancer patients with abnormal SNHG5 expression

Clinicopathologic characteristics	No. of studies	No. of patients	Odds ratio (95% CI)		Heterogeneity	
			Fixed	Random	*I*^*2*^(%)	*P*-value
Age	11	801	0.96 (0.71–1.29)	0.96 (0.71–1.30)	0	0.9
Gender	7	443	0.74 (0.49–1.12)	0.76 (0.46–1.25)	25	0.35
TNM (III + IV vs. I + II)	10	748	3.78 (2.75–5.19)	4.12 (2.62–6.46)	43	0.07
Digestive system	6	446	4.50 (2.98–6.8)	4.49 (2.96–6.81)	0	0.59
Reproductive system	3	262	2.36 (1.38–4.03)	2.79 (1.04–7.48)	59	0.09
Other system	1	40	16.00 (3.23–79.27)	16.00 (3.23–79.27)	–	–
LNM (present vs. absent)	8	638	2.16 (1.56–2.99)	2.16 (0.97–4.80)	80	< 0.0001
Digestive system	5	376	2.24 (1.48–3.38)	2.13 (0.59–7.64)	87	< 0.0001
Non-digestive system	3	262	2.03 (1.20–3.44)	2.12 (0.85–5.27)	64	< 0.0001
Tumor size	7	490	3.10 (2.13–4.50)	3.23 (2.07–5.03)	24	0.24
Digestive system	4	228	4.11 (2.34–7.21)	4.11 (2.33–7.26)	0	0.55
Non-digestive system	3	262	2.47 (1.50–4.08)	2.67 (1.26–5.64)	51	0.13
Histological grade	6	498	1.85 (1.29–2.67)	1.84 (1.25–2.70)	7	0.37
Digestive system	5	376	1.95 (1.28–2.98)	1.96 (1.18–3.26)	23	0.27
Non-digestive system	1	122	1.59 (0.77–3.27)	1.59 (0.77–3.27)	–	–
DM (present vs. absent)	3	296	4.61 (2.53–8.39)	4.11 (2.23–7.58)	0	0.4
Depth of invasion	2	190	1.46 (0.83–2.58)	1.78 (0.35–9.00)	85	0.009

TNM: Tumor Node Metastasis, LNM: lymph node metastasis, DM: distant metastasis, CI confidence interval,

No.: number, NA: not applicable, Random: Random effect model; Fixed: Fixed effect model; *I*^2^: I-square

### The correlation between *SNHG5* expression and LNM

**Fig. 4:**
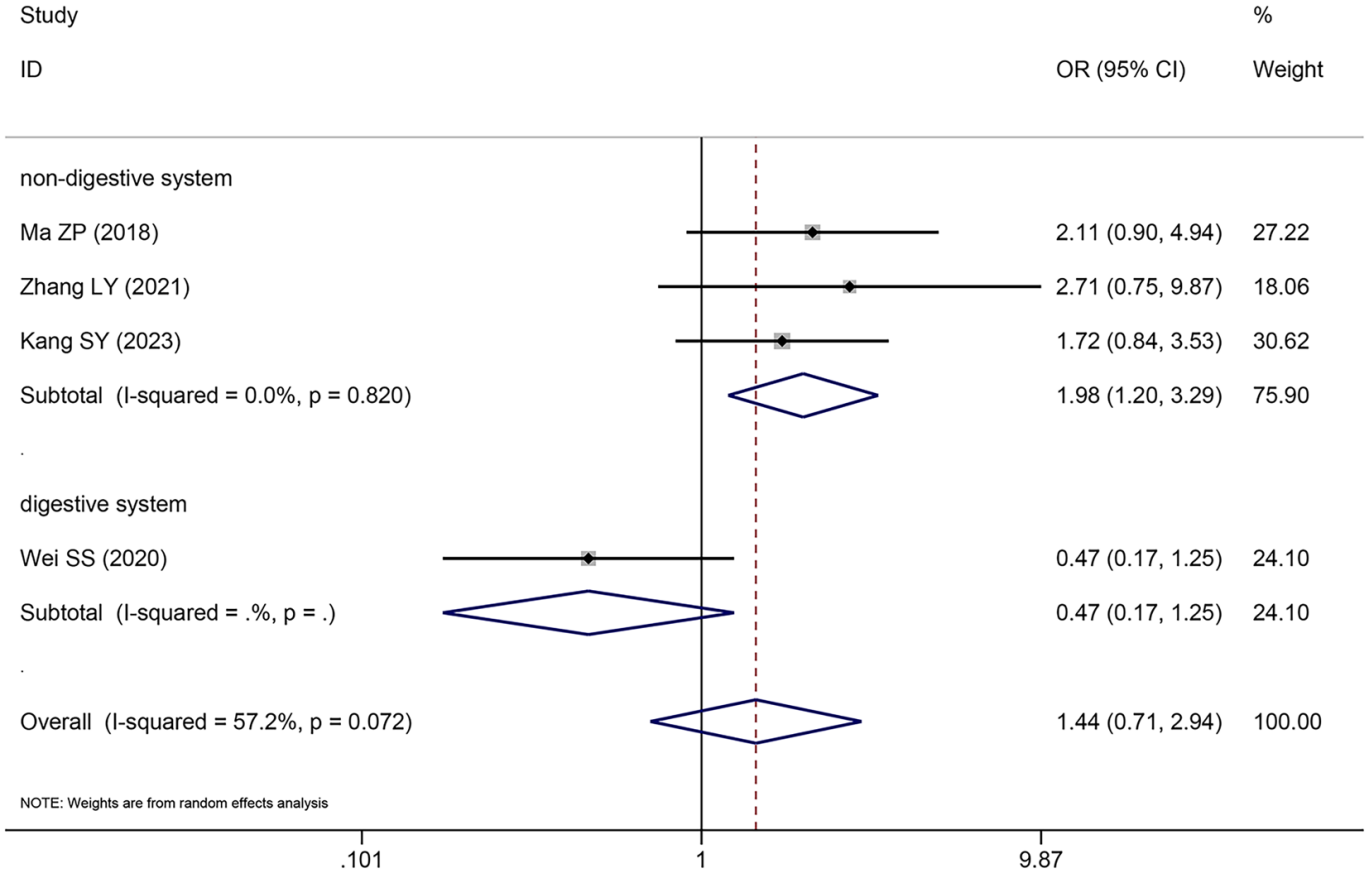
Forest plot of SNHG5 expression and LNM in cancers

Four papers with 270 cases were obtained in this analysis to assess the correlation between *SNHG5* expression and LNM. Combining the odds ratio (OR) with the 95% confidence interval (CI) indicated that *SNHG5* expression was positively related to LNM (OR: 1.443, 95% CI 0.709–2.939) (Fig. 4); however, the correlation was nonsignificant. The results of the subgroup analysis revealed a noteworthy correlation between *SNHG5* expression and easier-to-lymph node metastasis in the subgroup of patients with a nondigestive system invasion (OR = 1.992, 95% CI 1.204–3.295) and a nonsignificant correlation in the subgroup of patients with a digestive system invasion (OR = 0.467, 95% CI 0.175–1.245) (Table 4).

### Correlations between *SNHG5* expression and various other clinicopathological factors

**Fig. 5:**
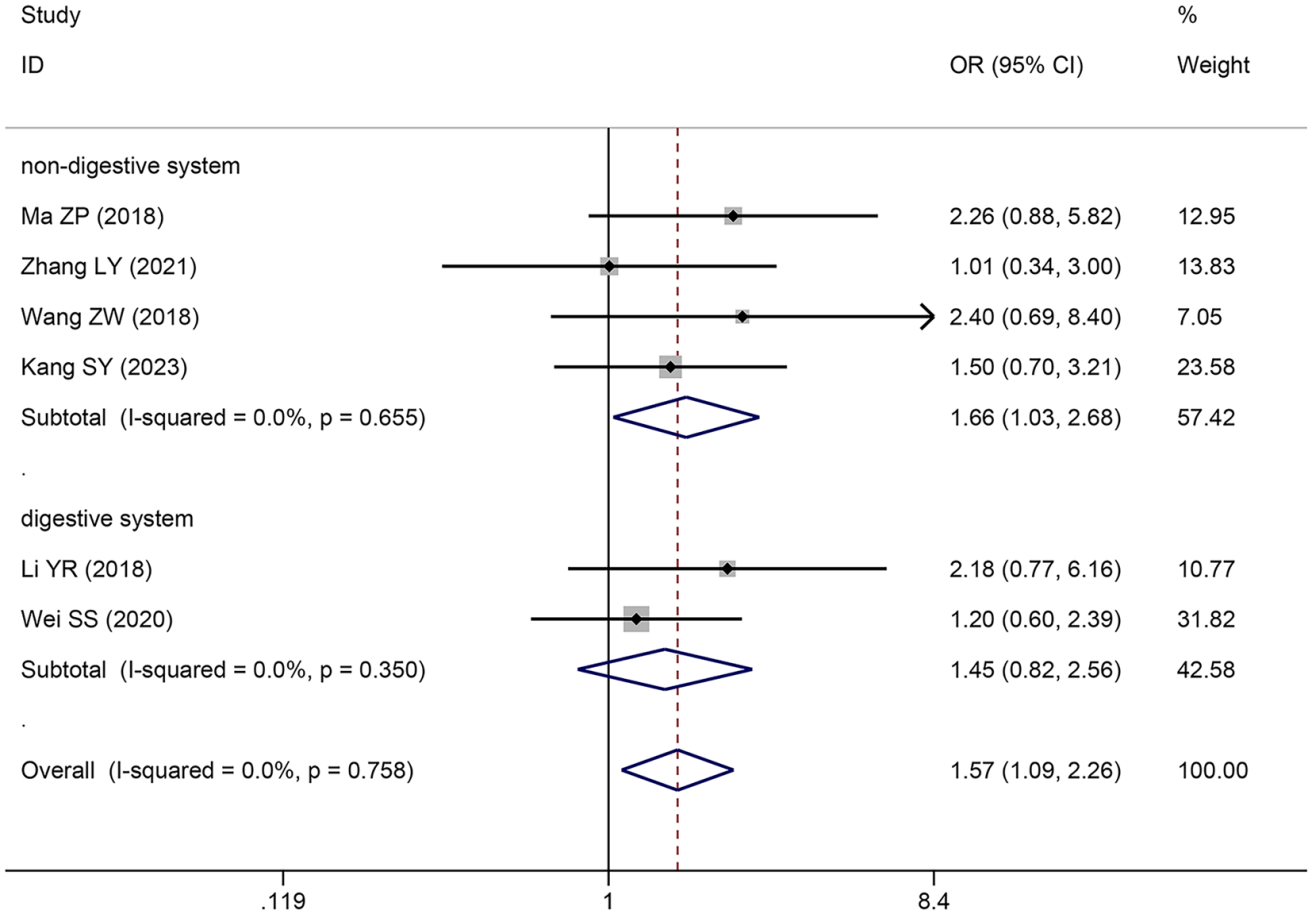
Forest plot of SNHG5 expression and tumor size in cancers

**Fig. 6:**
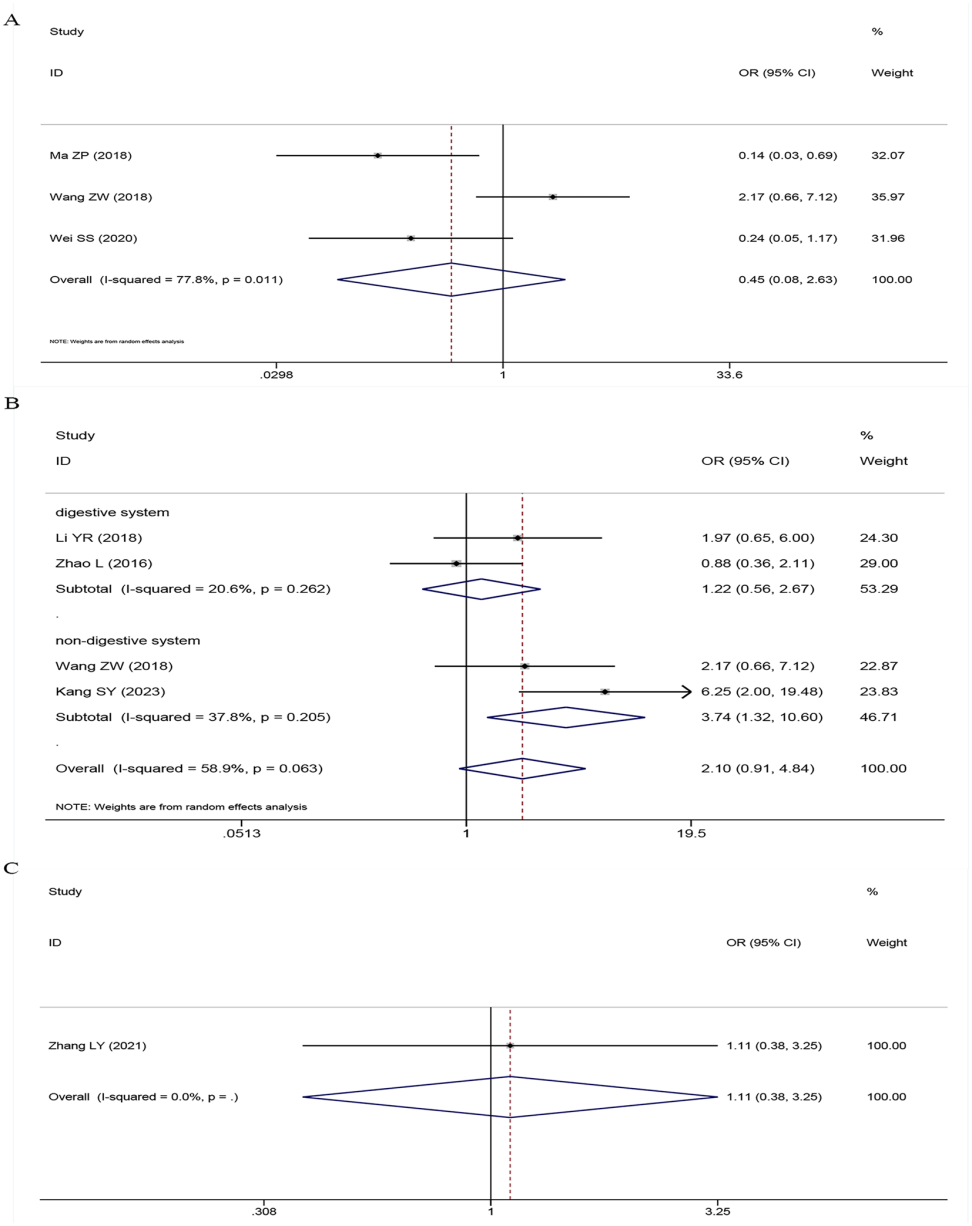
Forest plot of SNHG5 expression and DM, histological grade and depth of invasion in cancers. **A** DM; **B** histological grade; **C** depth of invasion

Analysis of the pooled ORs with 95% CIs revealed that elevated *SNHG5* expression was associated with increased tumour size (OR: 1.571, 95% CI 1.090–2.264) (Fig. 5); moreover, there were no significant associations between *SNHG5* expression and DM (OR: 0.449, 95% CI 0.077–2.630) (Fig. 6A), histological grade (OR: 2.098, 95% CI 0.910–4.838) (Fig. 6B), depth of invasion (OR: 1.106, 95% CI 0.376–3.248) (Fig. 6C), age (OR: 0.946, 95% CI 0.718–1.247) or sex (OR: 0.762, 95% CI 0.521–1.115) (Table 4).

### Sensitivity analysis and publication bias

**Fig. 7:**
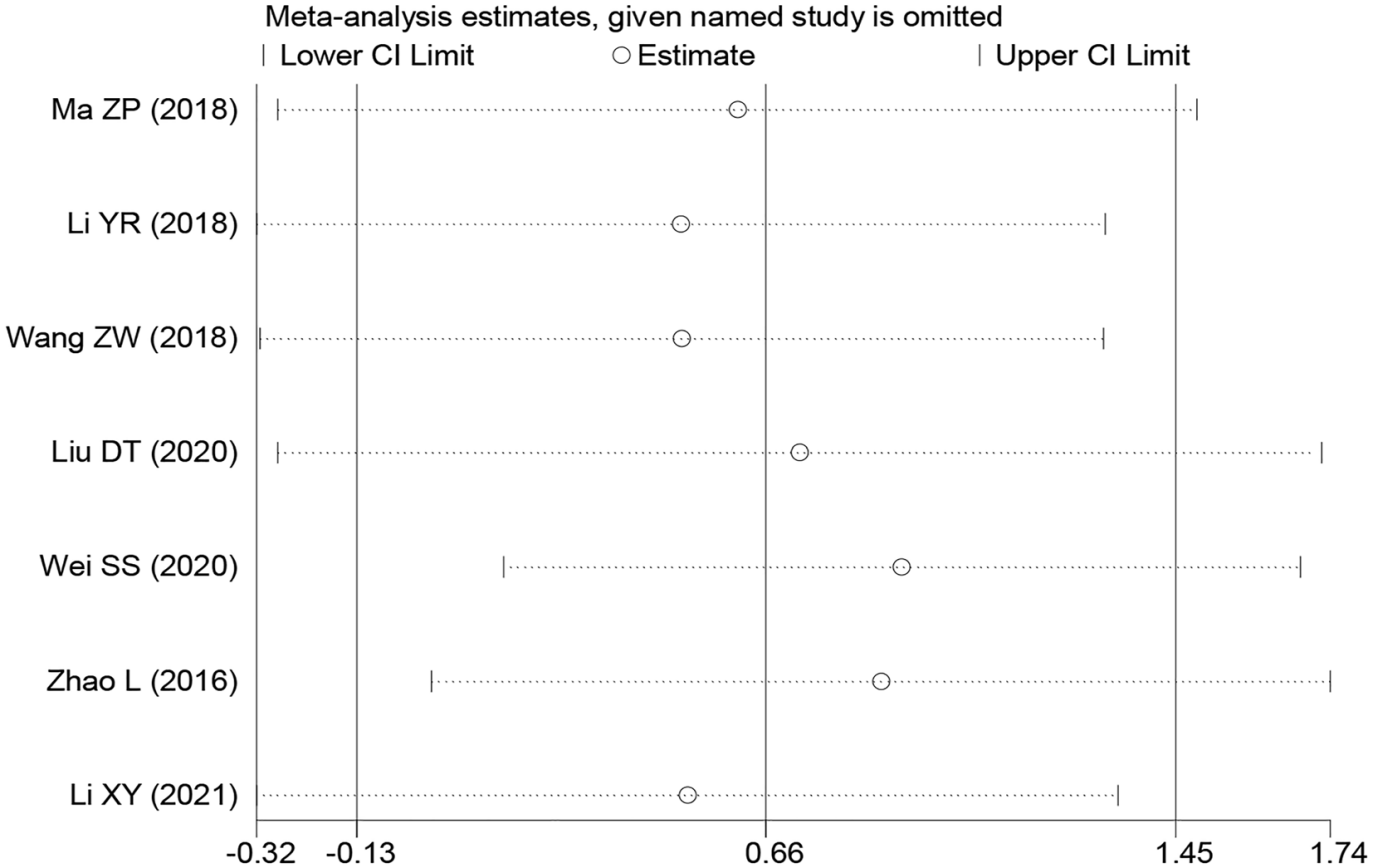
Sensitivity analysis for SNHG5 expression with overall survival (OS) in various cancers. HR: hazard ratio, CI confidence interval

**Fig. 8:**
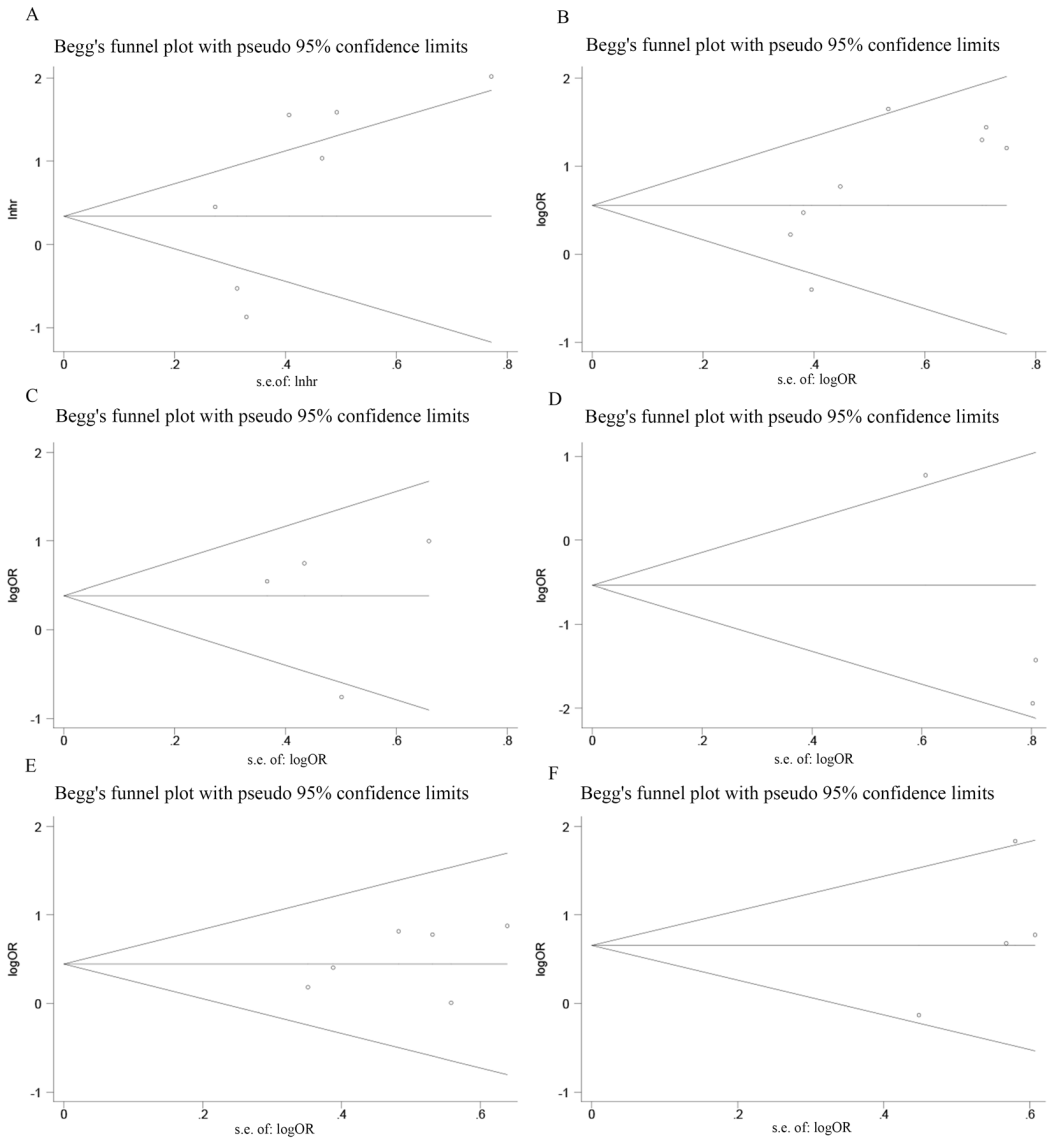
Beeg’s test about the relationship between SNHG5 expression and survival outcome in various cancers. **A** OS; **B** TNM stage; **C** LNM; **D** DM; **E** Tumor size; **F** Histological grade

The results of the sensitivity analysis of the overall survival rate showed that after removing the results of any one study, the overall survival was not affected at any time, suggesting that the overall survival rate was reliable and robust (Fig. 7). The results of Beeg's test of OS were as follows: Pr >|*z*|= 0.368, TNM stage = Pr >|*z*|= 0.108, LNM >|*z*|= 0.734, tumour size >|*z*|= 0.452, histological grade >|*z*|= 0.308, and DM >|*z*|= 1.000. These findings indicate that publication bias or other bias was not present in the original study (Fig. 8).

## Discussion

Cancer has consistently posed a profound threat to the wellbeing of humanity [1, 3]. Over the years, diverse treatment modalities have been incrementally employed to address this menace, leading to important advancements [5, 40]. Nevertheless, the survival outcomes in numerous cancers have reached a plateau, rendering further progress challenging. Consequently, there is an urgent need to explore innovative therapeutic strategies. Long noncoding RNAs have been confirmed to play a role in the emergence and progression of diverse ailments, including cardiovascular disease [41], metabolic disease [42], nervous system disease [43], rheumatic immune system disease and cancer [44]. Because cancer is the number one killer in human health, an increasing number of researchers have begun to uncover the underlying pathogenic mechanisms by which long noncoding RNAs (lncRNAs) contribute to cancer progression. Mounting evidence suggests that these RNAs can modulate key processes in tumour cells, including proliferation, migration, invasion, and apoptosis, and influence the response of these cells to chemotherapy and radiotherapy [45]. Long noncoding RNAs (lncRNAs) regulate stem cell transformation and epithelial-to-mesenchymal transition (EMT), and numerous such RNAs have been identified as potential targets for cancer therapeutics; these RNAs notably affect tumour progression and markedly predict tumour prognosis. Therefore, long noncoding RNAs are promising potential tumour therapeutic targets and prognostic markers.

This analysis included 11 original studies, and the scores assigned based on the NOS indicated a high level of research quality across all 11 documents. When the hazard ratio (HR) was combined, it was revealed that elevated expression of *SNHG5* could predict poor cancer prognosis, but the results were not statistically significant, and additional relevant high-quality original studies are needed to further support the results and conclusions of this investigation. Furthermore, combined OR data indicated that elevated *SNHG5* expression was a predictor of advanced TNM staging, larger tumour size, easier distant metastasis, and poor histological grade. However, the correlations of *SNHG5* expression with cancer LNM, invasion depth, age and sex were not statistically significant. In summary, the number of studies included in this meta-analysis was small, and the insufficient sample size may explain the reason for the effect of some prognostic indicators not reaching statistical significance. The results of the sensitivity analysis suggested that the overall survival results were reliable and robust. Furthermore, Begg's test results indicated the absence of any significant publication bias or other biases in the original studies.

**Fig. 9:**
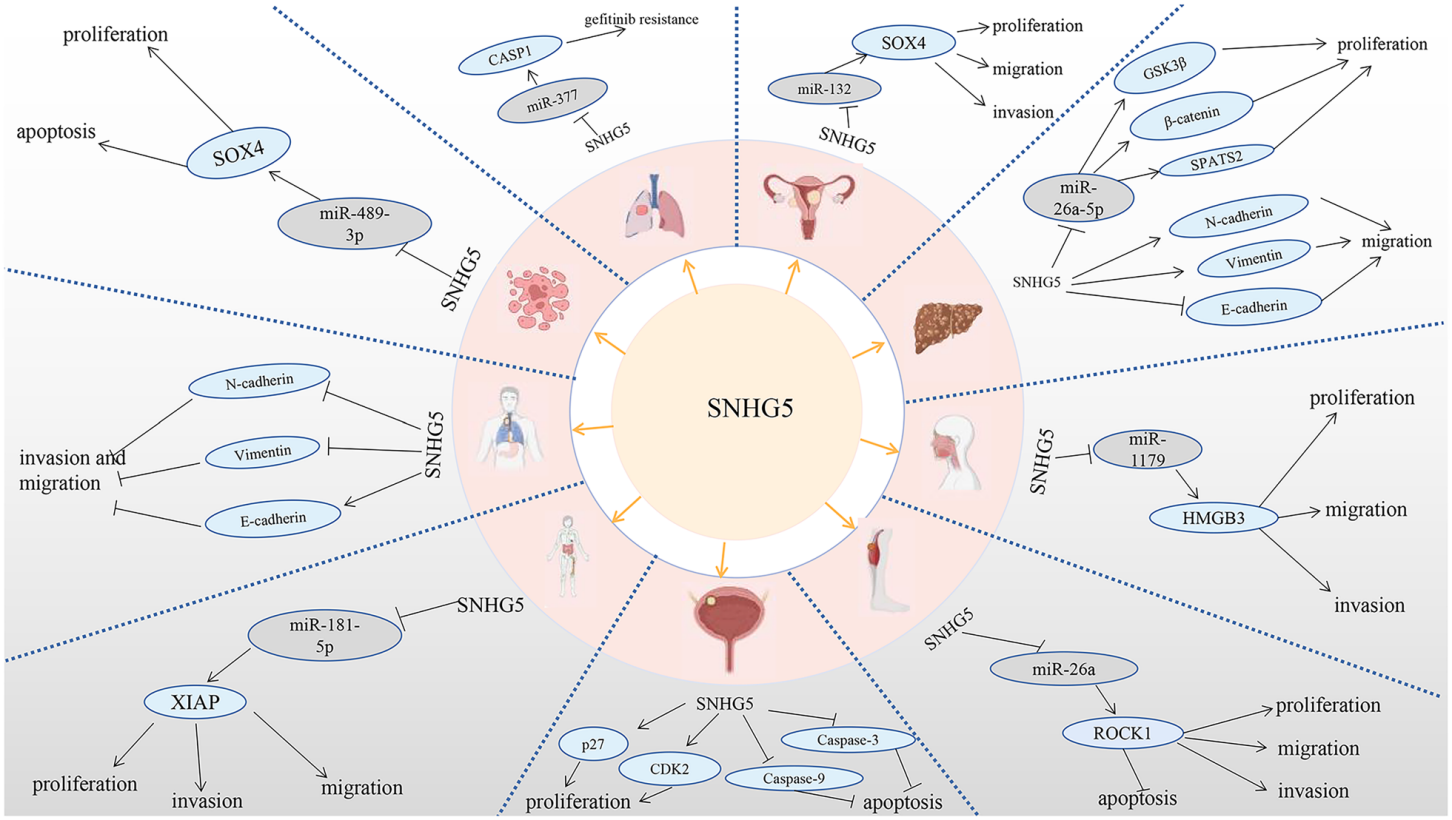
SNHG5 regulates some molecular biological mechanisms of tumor cells

*SNHG*5 was first revealed to be highly expressed as an oncogene in gastric cancer, and successive researchers subsequently reported that *SNHG5* was differentially expressed in bladder cancer, lung cancer, liver cancer, cervical cancer, osteosarcoma, laryngeal cancer, oral cancer, and lymphoma. An increasing number of researchers have explored the oncogenic mechanism of *SNHG5* (Fig. 9 and Table 5). Ma et al. reported that *SNHG5* contributes to proliferation and inhibits the apoptosis of bladder cancer cells by downregulating p27 and caspase-3 and caspase-9 and upregulating *CDK2* expression [32]. Wang et al. reported that *SNHG5* induces gefitinib resistance by upregulating its expression via the competitive sponging of miR-377 [46]. Li et al. discovered that *SNHG5* contributed to the proliferation and migration of hepatocellular carcinoma (HCC) cells through regulating GSK3β and the Wnt/β-catenin signalling pathway by competitively binding miR-26a-5p [33]. Yan et al. suggested that *SNHG5* could promote the proliferation and migration of HCC cells by upregulating spermatogenesis-associated serine-rich 2 (*SPATS2)* expression (47). Zhang et al. indicated that *SNHG5* accelerates the proliferation, migration and invasion of cervical cancer cells through upregulating sex-determining region Y-Box 4 (*SOX4*) expression via competitive sponging of miR-132 [34]. Wang et al. demonstrated that *SNHG5* could accelerate the migration, invasion and proliferation and inhibit the apoptosis of osteosarcoma cells through targeting and positively regulating Rho-associated coiled coil-containing protein kinase 1 (ROCK1) expression by sponging and negatively regulating miR-26a [35]. Liu et al. reported that *SNHG5* accelerated the proliferation, migration and invasion of nasopharyngeal carcinoma (NCC) cells by positively regulating high mobility group Box 3 (*HMGB3*) expression by downregulating miR-1179 expression [36]. Zhang et al. showed that *SNHG5* contributed to the proliferation—and inhibited apoptosis—of AML cells through accelerating sex-determining region Y-Box 4 (*SOX4*) expression by competitively binding to miR-489-3p [34]. Wei et al. discovered that *SNHG5* could inhibit the epithelial–mesenchymal transition (EMT) process in oesophageal cancer cells by downregulating the expression of metastasis-associated protein 2 (MTA2) [28]. Xing et al. revealed that *SNHG5* could promote proliferation, invasion, migration, and inhibited apoptosis of diffuse large B-cell lymphoma (DLBC) cells through the positive regulation of X-linked inhibitor of apoptosis protein (XIAP) expression via the competitive sponging of miR-181-5p [37]. Li et al. reported that high *SNHG5* expression indicated poor GC prognosis, but the detailed biological mechanism was not revealed [38]. Zhao et al. reported that *SNHG5* suppressed the proliferation, migration and invasion of gastric cancer cells by upregulating *MAT2* expression [39].

**Table 5:** Some molecular biological mechanisms of SNHG5 regulating cancer progression

Author	Year	Cancer type	Level of expression	MicroRNA	Gene	Function
Ma	2018	Bladder cancer	Upregulated	–	p27, caspase-3, caspase-9, CDK2	Induce proliferation, inhibit apoptosis
Wang	2018	Lung adenocarcinoma	upregulated	miR-377	CASP1	Gefitinib resistance
Li	2018	HCC	Upregulated	miR-26a-5p	GSK3β and Wnt/β-catenin signal pathway	Contributed to the proliferation and migration
Yan	2022	HCC	Upregulated	–	SPATS2	Induce the proliferation and migration
Zhang	2021	CC	Upregulated	miR-132	SOX4	Accelerate CC cell proliferation, migration and invasion
Wang	2018	osteosarcoma	Upregulated	miR-26a	ROCK1	Accelerate the migration, invasion and proliferation, inhibit apoptosis
Liu	2020	NCC	Upregulated	miR-1179	HMGB3	Drive cell proliferation, migration and invasion
Ying	2020	AML	Upregulated	miR-489-3p	SOX4	Contributed to the proliferation and inhibit the apoptosis
Wei	2021	esophageal cancer	Upregulated	–	MTA2	Inhibit the EMT process
Xing	2022	DLBCL	Upregulated	miR-181-5p	XIAP	Accelerated the proliferation, migration, and invasion

SNHG5: small nucleolar host gene 5; SPATS2: Spermatogenesis-associated serine-rich 2; CC: cervical cancer; SOX4: sex-determining region Y-box 4; NCC: nasopharyngeal Carcinoma; HMGB3: high mobility group box 3; AML: acute myeloid leukemia; EMT: Epithelial-mesenchymal transition; DLBCL: Diffuse Large B Cell Lymphoma; HCC: Hepatocellular carcinoma; "-": not reported

This study has several limitations. First, all patients in this meta-analysis were from China, and thus, the conclusions of this study are representative of only Asians. Second, the number of patients included in this study was insufficient, preventing some positive conclusions from reaching statistical significance. Third, some of the included studies did not provide hazard ratios (HRs) or 95% confidence intervals (95% CIs); therefore, we had to use the Engage software to analyze the survival data. This result is inconsistent with the data from the original SPSS. In addition, this study collected and explored only the relationship between *SNHG5* and the prognosis of some cancers, which may bias the results. However, additional high-quality studies with larger sample sizes are needed to support the conclusions of this study. Finally, several studies revealed that *SNHG5* is highly expressed in tumour cells, while others revealed that SNHG5 is expressed at low levels in tumour cells, leading to inconsistent experimental conclusions.

## Conclusion

*SNHG5* is abundantly expressed across numerous tumour tissues, and elevated *SNHG5* levels are significantly positively associated with poorer tumour prognosis. Additionally, high *SNHG5* expression predicts advanced TNM staging, increased susceptibility to distant metastasis, increased tumour diameter, and decreased histological grade. These findings suggest that *SNHG5* could emerge as a potential therapeutic target and a promising prognostic marker for tumour management. However, the conclusions drawn from this study require further validation through high-quality original research.

## Data Availability

All the data generated or analyzed throughout the course of this study have been included in the present publication. Additionally, any further data requests can be made to the corresponding author and will be provided upon reasonable request.
